# Occupational status and self-reported low back pain by gender: a nation-wide cross-sectional study among the general population in Japan

**DOI:** 10.1186/s12199-021-01031-2

**Published:** 2021-11-19

**Authors:** Kimiko Tomioka, Midori Shima, Keigo Saeki

**Affiliations:** Nara Prefectural Health Research Center, Nara Medical University, Kashihara, Nara, Japan

**Keywords:** Low back pain, Epidemiology, Cross-sectional studies, Occupation, Employment status, Japan

## Abstract

**Background:**

We aimed to examine the cross-sectional association between occupational class and self-reported low back pain (LBP) in a representative sample of the Japanese general population.

**Methods:**

We used anonymized data from a nationwide survey (31,443 men and 35,870 women aged ≥ 20). Occupational class variables included working status, occupation, employment status, and company size (number of employees). Covariates included age, socio-economic status, lifestyle, and comorbidities. Poisson regression models stratified by gender were used to estimate adjusted prevalence ratio (APR) and 95% confidence interval (CI) for self-reported LBP.

**Results:**

The prevalence of self-reported LBP was 11.7% in men and 14.2% in women. After adjustment for covariates and mutual adjustment for all occupational class variables, among both genders, agricultural/forestry/fishery workers and non-workers had a significantly higher prevalence of self-reported LBP: APR (95% CI) of agricultural/forestry/fishery was 1.36 (1.08–1.70) in men and 1.59 (1.30–1.93) in women; that of non-workers was 1.42 (1.18–1.70) in men and 1.23 (1.08–1.40) in women. Among men, non-regular employees were more likely to have self-reported LBP than regular employees: APR (95% CI) was 1.25 (1.07–1.46) in part-timers and casual staff and 1.18 (1.03–1.35) in other types of non-regular employees. Moreover, compared to men working at companies with ≥ 100 employees, men working at companies with 30–99 employees had a significantly higher prevalence of self-reported LBP (APR 1.17; 95% CI, 1.03–1.34). Among women, professionals and technicians (1.26; 1.11–1.43) and sales workers (1.22; 1.04–1.43) had a significantly higher prevalence of self-reported LBP than clerks. Neither employment status nor company size was associated with self-reported LBP in women. After stratified analyses by age group, similar patterns were observed in participants aged 20–64, but not in those aged ≥ 65.

**Conclusion:**

Our results suggest that self-reported LBP is highly prevalent among agricultural/forestry/fishery workers and the unemployed, regardless of gender, and that there are also gender differences in the association of occupational class factors with self-reported LBP. It is necessary, therefore, to take preventive measures against LBP based on gender and occupational class factors in Japan.

**Supplementary Information:**

The online version contains supplementary material available at 10.1186/s12199-021-01031-2.

## Background

Low back pain (LBP) is the leading cause of years lived with disability worldwide [1]. In Japan, the proportion of persons with subjective symptoms of LBP is about 10% of the population, ranking first among the male population and second only to shoulder stiffness in the female population [2]. For that reason, LBP is considered to be a common and urgent health problem in the Japanese general population.

Globally, 37% of all cases of LBP is caused by occupational risk factors, such as handling heavy objects, long hours in the same posture, unnatural postures, and vehicle driving work [3, 4]. In Japan, according to statistics on work-related illnesses from the Ministry of Health, Labour and Welfare, 8310 cases of work-related illnesses requiring a leave of absence of 4 days or more were reported in 2019, of which LBP cases accounted for 62.2% [5]. In other words, LBP is the most common occupational illness in Japan. However, these official workplace accident statistics only account for those who have industrial accident compensation insurance. Therefore, occupational LBP in workers who are not covered by workers' accident compensation insurance (e.g., sole proprietors) is unknown.

Many Japanese researchers have reported occupational LBP among hospital nurses [6], caregivers [7], construction workers [8], and taxi drivers [9]. However, these studies [6–9] have some limitations. First, previous studies targeted workers and did not include unemployed people. Epidemiological studies of occupational groups may underestimate the association between occupational risk factors and LBP due to the healthy worker effect [10]. In particular, women experience higher levels of unemployment than men [11], so it is important to include unemployed people in order to make a comparison. Second, because previous studies failed to conduct gender-specific analyses, it is unclear whether occupational LBP is the same or different for men and women.

In Japan, with its declining birthrate and aging population, the labor force is aging [11], and it is necessary to include people aged 65 and over when examining the relationship between occupational class variables and LBP. However, most previous studies assessing occupational LBP focused on those aged 64 and under and did not include those aged 65 and over [6–9, 12, 13]. There has been a large-scale population-based cohort study in Japan aimed at preventing musculoskeletal disorders [14]. This is an excellent study [14] of the general population including people over the age of 65 and using objective measurements such as X-ray radiography and bone mineral density measurement. However, it lacks occupational information.

By evaluating the relationship between occupational class variables and LBP in the sample from the entire population, including the uninsured workers, the unemployed, and older people, our study may provide basic data for LBP prevention measures that are important for the achievement of a healthy and long-lived society in Japan. Therefore, we aimed to investigate a gender-specific association of occupational class variables such as working status, occupation, employment status, and company size (number of employees) with self-reported LBP, using data from a nationwide cross-sectional survey in Japan.

## Methods

### Data and study participants

**Fig. 1:**
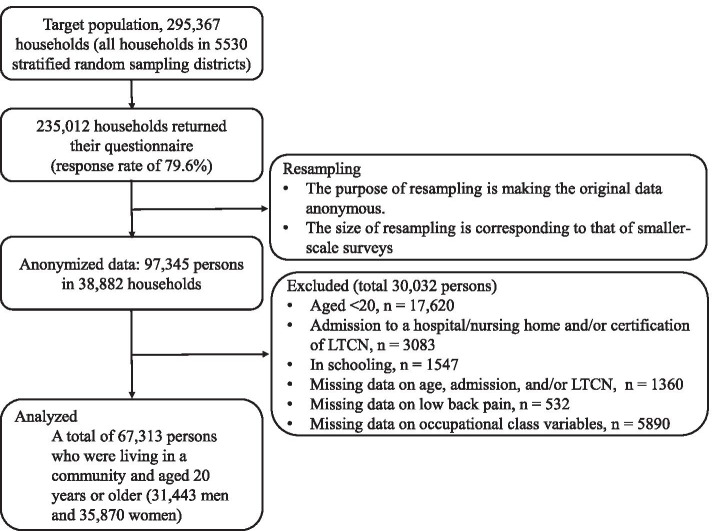
Selection of study participants. *LTCN* long-term care need

We used anonymized data from the 2013 Comprehensive Survey of Living Conditions (CSLC) conducted by the Ministry of Health, Labour and Welfare of Japan [2]. The CSLC is a nationally representative sample of the Japanese population, and the 2013 CSLC is the latest data available at the end of May, 2021. The details of the 2013 CSLC are explained elsewhere [15]. Briefly, the 2013 CSLC targeted all households (approximately 300,000 households) and household members (approximately 740,000 persons) in 5530 districts stratified and randomly selected from the 2010 census ward. Anonymized data had various anonymization measures (i.e., resampling). For example, about one-sixth of the data from the original CSLC was re-extracted, the age was given a 5-year-old class code, and the prefecture information was deleted. In the 2013 CSLC, people aged 19 and younger did not need to answer questions about drinking or smoking habits, and people in need of nursing care and those in a hospital/facility were exempt from answering about their health status and lifestyle. Therefore, among 97,345 anonymized data, we excluded 30,032 persons from our analyses because of being aged 20 or younger, being in a hospital/facility, being in school, having received long-term care certification, and missing data on age, hospital admission, working status, and/or self-reported LBP. The final number of participants included in this study was 67,313 persons (31,443 men and 35,870 women) (Fig. 1). The survey date was 6 June, 2013, but the work situation was as at May 2013.

### Measurements

#### Self-reported low back pain

The CSLC asked the respondents about their health status, “Have you been feeling sick (subjective symptoms) due to illness or injury in the last few days?” For this question, the respondents chose either “yes” or “no”. Those who answered that they had subjective symptoms selected all the applicable symptoms from 42 options. One of these 42 options included low back pain (LBP). In this study, those who chose LBP as a subjective symptom were defined as persons with self-reported LBP.

#### Occupational class variables

With reference to prior Japanese studies [16, 17], we adopted working status, occupation, employment status, and company size as occupational class variables.

Working status in this study was evaluated using the answer to the question, “Did you have any paid work during May of 2013?” A respondent who answered “Yes” was considered to be working. On the other hand, a respondent who answered “No” was considered to be non-working.

The CSLC asked persons with paid work about the type of job (hereafter, occupation) and whether they were employed or self-employed. Then, those who answered “employees” were asked about the labor force status as classified by the employer and the number of employees in their business establishment (hereafter, company size). Employed persons were asked to answer questions about their employment contract with seven response options (regular employees, part-timers, casual staff, temporary employees, contract staff, contract-based workers, fixed-term employees, and others); and company size with nine response options (1–4, 5–29, 30–99, 100–299, 300–499, 500–999, 1000–4999, 5000 or more, or public offices).

Occupation was separated into 12 groups based on the definition of the major classification of the Japanese Standard Occupational Classification (JSCO) [18]: managers; professionals and technicians; clerks; sales workers; services workers; security/protective workers; agricultural/forestry/fishery workers; manufacturing workers; transportation/machine workers; construction/mining workers; carrying/cleaning/packing workers; and other unclassified occupations. The JSCO has an adequate validity as a theory-based classification system suitable for evaluating Japan's occupation-related social status [19].

Employment status was classified into four groups: regular employees, part-timers and casual staff, other types of non-regular employees including temporary employees, contract staff, contract-based workers, and fixed-term employees, and the self-employed and others [16].

Company size was classified into six groups: 1–4, 5–29, 30–99, 100, or more, and public servants. The CSLC did not ask self-employed people about the size of their establishment. According to a survey of 5000 self-employed people nationwide [20], 98.2% of self-employed people answered that the number of employees was 4 or less. Therefore, in this study, company size of self-employed people was classified into 1 to 4 employees.

### Covariates

According to previous studies [4, 12, 21–23], the following variables were included as covariates that may be potential confounders of the association between LBP and occupational class: age, socio-economic status (SES), lifestyle habits, and chronic medical conditions. SES included marital status, family size, housing tenure, equivalent household expenditures, and education. Lifestyle habits included alcohol intake, smoking status, and sleep duration.

Marital status was categorized into married, never-married, and widowed/divorced. Family size was categorized into 1, 2, 3–4, and > 4. Housing tenure was dichotomized as owner-occupiers versus renters. Equivalent household expenditures (Japanese thousand yen per month) were divided into three groups by the tertiles (i.e., low, middle, and high), and this grouping by the tertiles was carried out by gender. Education (years of schooling) was categorized into < 10, 10–12, 13–15, and > 15. Alcohol intake (frequency of drinking) was categorized into none, several days a month, 1–4 days a week, and > 4 days a week. Smoking status was categorized into never-smokers, ex-smokers, and current smokers. Sleep duration (sleeping hours per day) was categorized into < 6, 6–7, and > 7. Chronic medical conditions were defined as persons with at least one disease under treatment for hypertension, diabetes mellitus, cerebrovascular disease, heart disease, and cancer.

Regarding the handling of missing values of the covariates, a group with missing values was created and included in the analysis subjects. By using this method, the influence of no answer on the covariates can be considered, and the number of analyzed participants can be maintained [24].

### Statistical analysis

Data comparisons between men and women or between those with and without self-reported LBP were tested using the chi-squared test for categorical variables and the t-test for continuous variables.

To investigate the cross-sectional association between occupational class variables and self-reported LBP, we used the generalized estimating equations of the multivariable Poisson regression model. The independent variables were occupation, employment status, and/or company size. Using clerks, the regular employees, or the companies with ≥ 100 employees as a reference, a prevalence ratio (PR) and a 95% confidence interval (CI) for self-reported LBP was calculated for other groups of occupation, employment status, or company size, respectively. In model 1, the age-adjusted PR was calculated. In model 2, all covariates (i.e., age, SES, lifestyle habits, and chronic medical conditions) were simultaneously added and the multivariate-adjusted PR was calculated. In model 3, to assess the independent association of each occupational class variable with self-reported LBP, we conducted mutual adjustment for all three items of occupational class variables, in addition to adjustment for all covariates.

Since LBP prevalence and occupational class variables vary by gender [4, 11], we performed stratified analyses by gender. The level of significance was 0.05 (two-tailed test). Statistical analyses were performed using the IBM SPSS Statistics Ver. 27 for Windows (Armonk, New York).

### Ethics

Based on Article 36 of the Statistics Act, we received approval of use for academic purposes from the Japanese Ministry of Health, Labour and Welfare (approval number 17003), and were provided with data without any information that would identify individuals.

## Results

### Characteristics of the study participants

The prevalence of individuals with self-reported LBP during the past few days was 13.0% (men 11.7%; women 14.2%), showing a significant gender difference (*P* < 0.001). The prevalence of unemployed women (49.8%) was about twice as high as that of men (26.1%), showing a significant gender difference (*P* < 0.001). For covariates, all variables differed significantly by gender (Table 1).

**Table 1:** Characteristics of the study participants by gender

		Men (*n* = 31,443)	Women (*n* = 35,870)	*P*-value^a^
Age: 65 years or older	*n* (%)	8802 (28.0)	11,236 (31.3)	< 0.001
Marital status: married	*n* (%)	22,533 (71.7)	23,558 (65.7)	< 0.001
Family size: one (i.e., living alone)	*n* (%)	3738 (11.9)	4079 (11.4)	0.037
Housing tenure: renters	*n* (%)	8434 (26.8)	9215 (25.7)	0.001
Household expenditures^b^	mean ± SD	14.78 ± 8.03	14.50 ± 7.91	< 0.001
Education: < 10 years of schooling	*n* (%)	4179 (13.3)	5319 (14.8)	< 0.001
Alcohol intake: > 4 days a week	*n* (%)	11,711 (37.2)	3924 (10.9)	< 0.001
Smoking status: current smokers	*n* (%)	10,677 (34.0)	3752 (10.5)	< 0.001
Sleep duration: < 6 h a day	*n* (%)	10,732 (34.1)	13,868 (38.7)	< 0.001
Chronic medical conditions^c^: present	*n* (%)	6905 (22.0)	6,532 (18.2)	< 0.001
Self-reported LBP: present	*n* (%)	3670 (11.7)	5,101 (14.2)	< 0.001
Work status: not working	*n* (%)	8204 (26.1)	17,869 (49.8)	< 0.001

*LBP* low back pain, *SD* standard deviation

^a^*P* values from chi-squared test for categorical variables and *t* test for continuous variables

^b^Monthly equivalent household expenditures (unit: Japanese one-thousand yen)

^c^Chronic medical conditions included hypertension, diabetes mellitus, cerebrovascular disease, heart disease, and cancer

In both genders, compared to persons without self-reported LBP, individuals with self-reported LBP were significantly older, more likely to have a low education, to be sleep deprived, and to have chronic medical conditions, while they were less likely to be working. Housing tenure did not differ between the two groups, regardless of gender (Table 2).

**Table 2:** Participant characteristics according to the presence or absence of self-reported LBP, by gender

		Men (*n* = 31,443)			Women (*n* = 35,870)		
		No LBP	LBP	*P* value^a^	No LBP	LBP	*P* value^a^
		(*n* = 27,773)	(*n* = 3670)		(*n* = 30,769)	(*n* = 5101)	
Age: 65 years or older	*n* (%)	7184 (25.9)	1618 (44.1)	< 0.001	8894 (28.9)	2342 (45.9)	< 0.001
Marital status: married	*n* (%)	19,673 (70.8)	2860 (77.9)	< 0.001	20,362 (66.2)	3196 (62.7)	< 0.001
Family size: one (i.e., living alone)	*n* (%)	3314 (11.9)	424 (11.6)	0.515	3306 (10.7)	773 (15.2)	< 0.001
Housing tenure: renters	*n* (%)	7498 (27.0)	936 (25.5)	0.057	7870 (25.6)	1345 (26.4)	0.233
Household expenditures^b^	mean ± SD	14.78 ± 8.04	14.76 ± 7.94	0.882	14.46 ± 7.88	14.78 ± 8.06	0.008
Education: < 10 years of schooling	*n* (%)	3476 (12.5)	703 (19.2)	< 0.001	4170 (13.6)	1149 (22.5)	< 0.001
Alcohol intake: > 4 days a week	*n* (%)	10,245 (36.9)	1466 (39.9)	< 0.001	3379 (11.0)	545 (10.7)	0.529
Smoking status: current smokers	*n* (%)	9499 (34.2)	1178 (32.1)	0.012	3142 (10.2)	610 (12.0)	< 0.001
Sleep duration: < 6 hours a day	*n* (%)	9351 (33.7)	1381 (37.6)	< 0.001	11,513 (37.4)	2355 (46.2)	< 0.001
Chronic medical conditions: present^c^	*n* (%)	5598 (20.2)	1307 (35.6)	< 0.001	5052 (16.4)	1480 (29.0)	< 0.001
Work status: non-working	*n* (%)	6776 (24.4)	1428 (38.9)	< 0.001	14,909 (48.5)	2960 (58.0)	< 0.001

*LBP* low back pain, *SD* standard deviation

^a^*P* values from chi-squared test for categorical variables and t-test for continuous variables

^b^Monthly equivalent household expenditures (unit: Japanese one-thousand yen)

^c^Chronic medical conditions included hypertension, diabetes mellitus, cerebrovascular disease, heart disease, and cancer

### Association between occupational class variables and self-reported LBP

After adjustment for all covariates (model 2), among men, agricultural/forestry/fishery workers and non-working people had a significantly higher PR for self-reported LBP than clerks. Regarding employment status, all types of non-regular employees and the self-employed had a significantly higher PR for self-reported LBP than regular employees. Regarding company size, workers who worked at companies with 1 to 4 employees and workers at companies with 30 to 99 employees had a significantly higher PR of self-reported LBP than those at companies with ≥ 100 employees (Table 3). Among women, a significant association was observed in professionals and technicians, sales workers, services workers, agricultural/forestry/fishery workers, and the non-working, compared to clerks. Regarding employment status, the self-employed had a significantly higher PR for self-reported LBP than regular employees. Regarding company size, it was not associated with self-reported LBP (Table 4).

**Table 3:** PRs (95% CIs) for self-reported LBP according to occupational class variables in 31,443 men

	*n*	% LBP	Model 1	Model 2	Model 3
			Age-adjusted	Multivariate-adjusted^a^	Mutually adjusted^b^
Occupation					
Clerks	1737	8.7%	1.00	1.00	1.00
Managers	2608	8.9%	0.89 (0.73–1.08)	0.86 (0.71–1.05)	0.85 (0.70–1.04)
Professionals and technicians	6084	8.5%	0.97 (0.82–1.16)	0.96 (0.81–1.14)	0.94 (0.79–1.12)
Sales	1846	8.7%	0.99 (0.80–1.22)	0.97 (0.79–1.20)	0.94 (0.76–1.17)
Services	2925	8.8%	0.96 (0.79–1.16)	0.93 (0.77–1.12)	0.88 (0.72–1.07)
Security/protective	505	9.7%	0.99 (0.73–1.36)	0.95 (0.70–1.30)	0.93 (0.68–1.26)
Agricultural/forestry/fishery	993	12.2%	1.36 (1.11–1.68)	1.42 (1.15–1.75)	1.36 (1.08–1.70)
Manufacturing	2215	11.3%	1.13 (0.93–1.38)	1.09 (0.89–1.33)	1.06 (0.86–1.30)
Transportation/machine	993	9.1%	1.28 (1.02–1.61)	1.17 (0.93–1.47)	1.13 (0.89–1.42)
Construction/mining	1713	17.0%	1.21 (0.99–1.49)†	1.17 (0.96–1.44)	1.12 (0.91–1.39)
Carrying/cleaning/packing	775	11.5%	1.19 (0.93–1.52)	1.12 (0.87–1.44)	1.04 (0.80–1.34)
Other unclassified occupation	845	10.8%	1.10 (0.86–1.40)	1.12 (0.88–1.43)	1.05 (0.82–1.34)
Non-working	8204	17.4%	1.27 (1.08–1.50)	1.29 (1.09–1.53)	1.42 (1.18–1.70)
Employment status					
Regular employees	14,249	8.2%	1.00	1.00	1.00
Part-timers and casual staff	1446	12.1%	1.22 (1.05–1.42)	1.26 (1.08–1.46)	1.25 (1.07–1.46)
Other types of non-regular^c^	1862	12.0%	1.18 (1.03–1.35)	1.19 (1.03–1.36)	1.18 (1.03–1.35)
Self-employed	5682	11.9%	1.11 (1.01–1.22)	1.15 (1.04–1.26)	1.12 (0.88–1.42)
Non-working	8204	17.4%	1.31 (1.19–1.44)	1.38 (1.24–1.52)	Not calculated
Company size (number of employees)					
≥ 100 employees	9288	8.3%	1.00	1.00	1.00
1 to 4 employees	6356	11.7%	1.10 (0.999–1.22)	1.13 (1.02–1.25)	1.02 (0.80–1.29)
5 to 29 employees	3211	9.5%	1.08 (0.95–1.22)	1.06 (0.93–1.21)	1.02 (0.89–1.16)
30 to 99 employees	2796	10.4%	1.20 (1.06–1.37)	1.20 (1.06–1.37)	1.17 (1.03–1.34)
Public servants	1588	8.1%	0.94 (0.78–1.12)	0.97 (0.81–1.16)	0.99 (0.82–1.19)
Non-working	8204	17.4%	1.29 (1.16–1.42)	1.34 (1.20–1.49)	Not calculated

*CI* confidence interval, *LBP* low back pain, *PR* prevalence ratio

^a^Adjusted for age (per 5-year increase), marital status, family size, housing tenure, equivalent household expenditures, education, alcohol intake, smoking status, sleep duration, and chronic medical conditions

^b^In addition to model 2, all three items of occupational class variables were included (i.e., Model 3 was mutually adjusted for all occupational class variables)

^c^Temporary employees, contract staff, contract-based workers, and fixed-term employees

**Table 4:** PRs (95% CIs) for self-reported LBP according to occupational class variables in 35,870 women

	*n*	% LBP	Model 1	Model 2	Model 3
			Age-adjusted	Multivariate-adjusted^a^	Mutually adjusted^b^
Occupation					
Clerks	4260	9.4%	1.00	1.00	1.00
Managers	359	10.0%	0.87 (0.63–1.20)	0.88 (0.64–1.22)	0.87 (0.63–1.21)
Professionals and technicians	4110	11.9%	1.26 (1.11–1.42)	1.26 (1.11–1.42)	1.26 (1.11–1.43)
Sales	1687	13.0%	1.29 (1.10–1.50)	1.25 (1.07–1.46)	1.22 (1.04–1.43)
Services	4357	12.2%	1.20 (1.06–1.35)	1.14 (1.01–1.29)	1.12 (0.98–1.27)
Security/protective	16	6.3%	0.73 (0.11–4.95)	0.69 (0.10–4.84)	0.67 (0.10–4.70)
Agricultural/forestry/fishery	613	21.9%	1.52 (1.27–1.82)	1.63 (1.35–1.95)	1.59 (1.30–1.93)
Manufacturing	1172	11.8%	1.14 (0.95–1.37)	1.09 (0.91–1.31)	1.07 (0.89–1.29)
Transportation/machine	30	16.7%	1.64 (0.75–3.60)	1.39 (0.62–3.12)	1.37 (0.61–3.06)
Construction/mining	78	14.1%	1.30 (0.74–2.29)	1.27 (0.71–2.27)	1.25 (0.70–2.24)
Carrying/cleaning/packing	555	14.8%	1.28 (1.03–1.60)	1.17 (0.94–1.46)	1.13 (0.90–1.42)
Other unclassified occupation	764	12.4%	1.16 (0.94–1.43)	1.13 (0.92–1.40)	1.11 (0.90–1.37)
Non-working	17,869	16.6%	1.19 (1.08–1.33)	1.20 (1.08–1.33)	1.23 (1.08–1.40)
Employment status					
Regular employees	6297	9.8%	1.00	1.00	1.00
Part-timers and casual staff	6495	12.3%	1.10 (0.99–1.21)	1.09 (0.98–1.21)	1.10 (0.99–1.23)
Other types of non–regular^c^	1687	11.6%	1.09 (0.94–1.27)	1.08 (0.93–1.26)	1.08 (0.93–1.26)
Self-employed	3522	14.9%	1.08 (0.97–1.21)	1.15 (1.02–1.28)	1.24 (0.98–1.57)
Non-working	17,869	16.6%	1.07 (0.98–1.17)	1.11 (1.01–1.22)	Not calculated
Company size (number of employees)					
≥100 employees	6617	11.3%	1.00	1.00	1.00
1 to 4 employees	4261	14.2%	0.96 (0.87–1.07)	1.02 (0.92–1.13)	0.85 (0.69–1.06)
5 to 29 employees	3411	11.1%	0.94 (0.83–1.05)	0.94 (0.84–1.06)	0.94 (0.84–1.06)
30 to 99 employees	2628	11.1%	0.95 (0.83–1.08)	0.95 (0.84–1.08)	0.95 (0.84–1.08)
Public servants	1084	11.3%	0.97 (0.81–1.16)	1.05 (0.88–1.26)	1.06 (0.88–1.28)
Non-working	17,869	16.6%	0.97 (0.89–1.05)	1.01 (0.92–1.10)	Not calculated

*CI* confidence interval, *LBP* low back pain, *PR* prevalence ratio

^a^Adjusted for age (per 5-year increase), marital status, family size, housing tenure, equivalent household expenditures, education, alcohol intake, smoking status, sleep duration, and chronic medical conditions

^b^In addition to model 2, all three items of occupational class variables were included (i.e., Model 3 was mutually adjusted for all occupational class variables)

^c^Temporary employees, contract staff, contract-based workers, and fixed–term employees

After mutual adjustment for all three items of occupational class variables (model 3), among men, the self-employed and small-sized companies with 1 to 4 employees lost their significance, while the association of agricultural/forestry/fishery workers (adjusted PR 1.36; 95% CI, 1.08–1.70), non-working people (adjusted PR 1.42; 95% CI, 1.18–1.70), part-timers and casual staff (adjusted PR 1.25; 95% CI, 1.07–1.46), other types of non-regular staff (adjusted PR 1.18; 95% CI, 1.03–1.35), and medium-sized companies with 30 to 99 employees (adjusted PR 1.17; 95% CI, 1.03–1.34) with self-reported LBP remained significant (Table 3). Among women, services workers and the self-employed lost their significance, while the association of professionals and technicians (adjusted PR 1.26; 95% CI, 1.11–1.43), sales workers (adjusted PR 1.22; 95% CI, 1.04–1.43), agricultural/forestry/fishery workers (adjusted PR 1.59; 95% CI, 1.30–1.93), and non-working people (adjusted PR 1.23; 95% CI, 1.08–1.40) with self-reported LBP remained significant. Among women, neither employment status nor company size was associated with self-reported LBP (Table 4). These results of the mutually adjusted model suggest that agricultural/forestry/fishery occupations were independent factors for self-reported LBP in both men and women, and that the association of self-employment and small-sized companies with self-reported LBP was confounded by the agricultural/forestry/fishery occupations.

Using the final model (model 3), we conducted additional analyses in which the participants were divided into two age groups (i.e., under 65 and over 65). After stratified analyses by age group, similar patterns were observed in participants aged from 20 to 64 years, but not in those aged 65 and over (Supplementary Tables 1 and 2).

## Discussion

The current study reveals high prevalence of self-reported LBP in agricultural/forestry/fishery workers and non-working people, regardless of gender. Employment status and company size were associated with self-reported LBP in men, while occupation was associated with self-reported LBP in women. To the best of our knowledge, this is the first study in Japan to demonstrate that agricultural/forestry/fishery workers are occupational groups at high risk of self-reported LBP and that there are gender differences in the association of occupational class variables with self-reported LBP.

Regarding occupation, we found an increased prevalence of self-reported LBP among agricultural/forestry/fishery workers in both genders. In countries other than Japan, many researchers have reported that agricultural/forestry/fishery workers are at high-risk of occupational LBP [13, 25, 26]. However, the subjects of previous studies have often been limited to males, and gender differences in occupational LBP among agricultural/forestry/fishery workers have not been examined. This study is the first report to show that not only men but also women are at high risk of self-reported LBP in agricultural/forestry/fishery work. In Japan, in terms of the 5132 cases certified as work-related LBP by industry [5], the largest number was in the health services industry including nurses and care workers, accounting for 32.1%, while the percentage of cases in agriculture/forestry/fishery industry was as low as 1.1%. The low percentage may be explained as follows. Many agricultural/forestry/fishery workers work as sole proprietors [20] (for example, among the participants in this study, nine out of ten agricultural/forestry/fishery workers are self-employed), and do not have workers' accident compensation insurance. Therefore, they are not captured in the industrial accident statistics. Risk factors for LBP in agriculture/forestry/fishery workers include physical exposures (e.g., heavy lifting, hard physical work, whole-body/hand-arm vibrations, and awkward postures) and environmental exposures such as extreme cold or heat [25, 26]. It is urgently necessary to take measures against LBP in agricultural/forestry/fishery workers in Japan.

Our results of female high prevalence of self-reported LBP in professionals and technicians are in line with previous research findings; LBP is a major issue among the working-age population, particularly among health care professionals who perform patient lifting and transfer tasks [27, 28]. However, these previous studies have not focused on gender differences in occupational LBP. In the JSCO, nurses are classified as professional workers [18]. It has been reported that nurses in numerous countries including Japan are predominantly women and are at high risk of LBP [6, 27–29]. Therefore, among women, professionals and technicians had a significantly higher PR for self-reported LBP than clerks, but these relationships were not found in men.

For female sales workers, we did not have access to previous studies reporting a high prevalence of LBP, but two reports suggest an elevated risk of LBP in sales workers. First, a cross-sectional survey in South Korea reported that cosmetics saleswomen were in a poorer physical and mental condition than general working women, because they are exposed to working long hours in a standing position and to violence from customers [30]. Second, a prior study on the smoking prevalence of Japanese reported that in 2013, the smoking prevalence of female sales workers was 17.8% (95%CI, 17.0–18.6), which was significantly higher than that of female clerical workers at 9.9% (95% CI, 9.5–10.3) [31]. Prolonged standing [32], workplace violence [33], and smoking [4, 21, 34] have been identified as risk factors for LBP, which may have led to a higher prevalence of self-reported LBP among Japanese female sales workers.

Regarding employment status, among men, non-regular employees including part-timers and temporary/contract workers had a significantly higher prevalence of self-reported LBP than regular employees, while among female workers, employment status was not associated with self-reported LBP. Our findings in men are concordant with previous research showing that migrant construction workers with precarious employment [35] or workers with non-standard work arrangements [13] had an increased risk for self-reported LBP. For women, a Japanese cross-sectional study of 21,450 workers aged 40–59 years reported that non-regular employees were less likely to report poor self-rated health compared with regular employees, and that a low probability of poor self-rated health among non-regular employees may be explained by their reduced level of work-family conflict [36]. Because work-family conflict is identified as one of work-related risk factors for self-reported LBP [13], Japanese female regular employees may experience work-family conflict and fail to get the LBP prevention effects of stable employment.

In this study, company size was significantly associated with self-reported LBP in men, but not in women. Several previous studies suggest that company size may affect self-reported LBP. A cross-sectional survey in South Australia reported that workers in medium-sized workplaces had a higher prevalence of musculoskeletal pain and discomfort than those in large companies [37]. A nationwide cross-sectional survey in France reported that employees in small-scale companies were more exposed to physically demanding working conditions [38], which is one of the risk factors for occupational LBP [3, 4]. However, in this study, the proportion of men with self-reported LBP was significantly higher in companies with 30 to 99 employees, but not in small-sized companies with 1 to 4 employees. In a previous study using a nationally representative sample in Japan [16], company size was associated with mental health only among men, and a non-linear relationship between company size and mental health was observed: males working at companies with 300 to 999 employees had significantly worse mental health than those at companies with 1 to 29 employees. Although company size is a potential risk factor for self-reported LBP, it is unclear why the association between company size and self-reported LBP was significant only for men and why there was no dose-response relationship. Further research is needed.

In this study, the prevalence of self-reported LBP was significantly higher for non-working people than for working people. This is consistent with a nationally representative survey in the USA [23]. The reason for the high prevalence of self-reported LBP among the non-working can be explained by the fact that some unemployed people are unable to work due to LBP [39], and that unpaid domestic work such as housework, childcare, and long-term care is a significant risk factor for LBP [34]. LBP prevention measures need to target not only the working population but also the non-working population.

Stratified analyses by age group revealed that the association between occupational class factors and self-reported LBP was due to that in people aged 20 to 64 years. There are two possible reasons as to why there is no association in people aged 65 and over. First, according to a Japanese official survey in 2017 [40], 93.4% of companies have a uniform retirement age; 79.3% of these companies have a retirement age of 60 and 17.8% have a retirement age of 65 or older. Regular employees who have reached retirement age are often rehired as non-regular employees [40]. Since the occupational class of employees depends on whether they have reached retirement age, the relationship between occupational class variables and self-reported LBP is considered to be largely influenced by age. Second, because the proportion of employed people over the age of 65 is low, the number of occupational class factors in workers is small, leading to a decline in the power to detect the association with LBP. The proportion of people aged 65 and over in the working population was 5.0% in 1985, but has continued to rise to 9.9% in 2013 and 13.4% in 2020 [41]. Workforce participation of older adults has been increasing year by year in recent years, and LBP in older workers is becoming a new occupational health problem. Therefore, further research needs to be carried out to clarify the association between occupational class factors and LBP in older adults.

There are two previous studies that estimated the prevalence of people with LBP in the general population. First, the LOCOMO study with 12,019 residents in nine communities of Japan evaluated LBP on most days (and continuously on at least one day) in the past month and found that the prevalence of LBP was 37.7% (men 34.2%; women 39.4%) [14]. Of the study participants, 67.1% were women and 60.6% were people aged 70 and over [14]. Because both female gender and higher age are risk factors for LBP [4, 21, 23], the prevalence of LBP in the LOCOMO study might be higher than that in our study. Second, the NHANES study with a nationally representative sample of the US population assessed LBP lasting almost every day for at least 3 months and reported that the prevalence of LBP in US adults aged 20–69 years old was 13.1% [23]. The prevalence of LBP was similar in the NHANES study and our study. It should be noted that the NHANES study had younger participants and a stricter definition of LBP compared to our study. Additionally, different LBP assessment questionnaires were used in each study. Although direct comparisons between studies are difficult, these results suggest that LBP prevalence in this study is not particularly low.

The strengths of this study are as follows: First, because the CSLC is a large-scale survey at the national level, it sufficiently guarantees the representativeness and generalization of Japanese people. Furthermore, by using the CSLC, we were able to examine the relationship of occupational class variables with self-reported LBP by gender. Second, because the CSLC gathered information on SES, lifestyle, and health status, we were able to use the covariates necessary for the relationship between occupational class factors and self-reported LBP.

This study has some limitations. First, because the study is a cross-sectional design, it is not possible to identify a causal relationship. People with self-reported LBP have the potential to leave a job or perform light duties. A longitudinal cohort study is needed to clarify the causal relationship between occupational class variables and self-reported LBP. Second, the CSLC asked respondents about the presence or absence of subjective symptoms of LBP and the status and content of their current work. Our results may have been affected by misclassification based on self-reporting and exclusion from analysis due to non-reporting. Misclassification may lead to a null association between occupational class factors and self-reported LBP [10]. The impact of non-responders not being analyzed may lead to an underestimation of the observed association, because people with low occupational status are more likely to not respond than those with high occupational status, and those with low occupational status are at high risk of LBP [10, 23]. Third, the definition of LBP in this study is LBP during the past few days. Previous studies often asked about LBP experience over the past year [4, 12, 22, 25, 26], and some studies asked about the degree and duration of LBP [4, 6, 8, 9, 26]. Because our LBP definition includes a shorter time period than that of previous studies, this may lead to a lower prevalence of LBP. On the other hand, because we failed to consider the degree and duration of pain, our LBP may include milder LBP than previous studies, which may result in a higher prevalence of LBP. Therefore, it is unknown whether the prevalence of people with LBP in this study is overestimated or underestimated compared to previous studies.

Regarding implications, our results help identify populations of high priority when considering measures aimed at reducing or preventing self-reported LBP in the people of Japan. Policymakers should take measures to prevent LBP by positioning agricultural/forestry/fishery workers as being in occupations with high risk of LBP, which has not been captured by public occupational accident statistics. Clinicians need to be aware of the working status and occupational class of patients who complain of LBP. In particular, they should also note whether LBP patients are unemployed, whether male patients are non-regular employees or workers of medium-sized companies, and what occupations female patients are engaged in. Moreover, recent studies have suggested that fitness for work interventions are effective for workers with LBP [42]. Therefore, regarding implications for the workplace, occupational physicians need to implement workplace measures that enhance fitness for work so that workers can work safely.

## Conclusions

Using a nationally representative sample, this study examined the cross-sectional relationship between occupational class variables and self-reported LBP in Japanese people by gender. Our results showed that the prevalence of self-reported LBP was significantly higher in agricultural/forestry/fishery workers and unemployed people for both men and women, independently of confounding factors such as age, SES, lifestyle, and comorbidities. In men, employment status and company size were significantly associated with self-reported LBP, while in women, occupation was significantly associated with self-reported LBP. Our findings indicate that it is urgently necessary to take measures targeting occupational LBP in agricultural/forestry/fishery workers who are missing from public occupational accident statistics, and that it is necessary and effective to provide an occupational LBP prevention program that takes gender differences into account.

## Supplementary Information

**Additional file 1: Supplementary Table 1.** Stratified analyses by age group among men. **Supplementary Table 2.** Stratified analyses by age group among women.

## Data Availability

Data are available from the Ministry of Health, Labour and Welfare, Japan (https://www.mhlw.go.jp/toukei/itaku/tokumei.html) for researchers who obtain approval to use the anonymous data in accordance with Article 36 of the Statistics Law of Japan.

## Abbreviations

CI: Confidence interval
CSLC: Comprehensive Survey of Living Conditions
JSCO: Japanese Standard Occupational Classification
LBP: Low back pain
PR: Prevalence ratio
SES: Socio-economic status
