# Supplementary material for: Occupational status and self-reported low back pain by gender: a nation-wide cross-sectional study among the general population in Japan
Source: Environ Health Prev Med. 2021 Nov 19;26:111. doi: 10.1186/s12199-021-01031-2 (PMC8603561; doi:10.1186/s12199-021-01031-2)
Supplement: Supplementary file 1 — Additional file 1: Supplementary Table 1. Stratified analyses by age group among men. Supplementary Table 2. Stratified analyses by age group among women. [file 12199_2021_1031_MOESM1_ESM.docx]

**Supplementary Table** 1 Stratified analyses by age group among men

|  |  | People aged 20–64 years (n = 22,641) | | |  | People aged 65 and older (n = 8,802) | | |
| --- | --- | --- | --- | --- | --- | --- | --- | --- |
|  |  | n | % LBP | PR^a^ (95% CI) |  | n | % LBP | PR^a^ (95% CI) |
| Occupation | |  |  |  |  |  |  |  |
|  | Clerks | 1654 | 8.1% | 1.00 |  | 83 | 20.5% | 1.00 |
|  | Managers | 2285 | 7.8% | 0.84 (0.68–1.04) |  | 323 | 16.7% | 0.81 (0.50–1.32) |
|  | Professionals & technicians | 5577 | 7.9% | 0.96 (0.80–1.16) |  | 507 | 15.2% | 0.74 (0.47–1.18) |
|  | Sales | 1637 | 7.9% | 0.99 (0.78–1.25) |  | 209 | 14.4% | 0.71 (0.42–1.22) |
|  | Services | 2494 | 8.1% | 0.95 (0.77–1.18) |  | 431 | 12.3% | 0.56 (0.34–0.90) |
|  | Security/protective | 449 | 6.5% | 0.72 (0.49–1.07) |  | 56 | 30.4% | 1.10 (0.62–1.97) |
|  | Agricultural/forestry/fishery | 479 | 12.9% | 1.38 (1.02–1.87) |  | 514 | 20.8% | 1.02 (0.64–1.64) |
|  | Manufacturing | 2050 | 9.4% | 1.15 (0.92–1.43) |  | 165 | 13.9% | 0.63 (0.36–1.11) |
|  | Transportation/machine | 876 | 12.3% | 1.28 (0.997–1.64) |  | 117 | 11.1% | 0.48 (0.25–0.94) |
|  | Construction/mining | 1507 | 10.7% | 1.16 (0.92–1.46) |  | 206 | 15.5% | 0.84 (0.49–1.43) |
|  | Carrying/cleaning/packing | 641 | 9.8% | 1.07 (0.80–1.44) |  | 134 | 19.4% | 0.77 (0.44–1.33) |
|  | Other unclassified occupation | 669 | 8.7% | 1.04 (0.77–1.39) |  | 176 | 18.8% | 0.88 (0.53–1.47) |
|  | Non-working | 2323 | 12.6% | 1.64 (1.32–2.03) |  | 5881 | 19.3% | 0.77 (0.47–1.26) |
| Employment status | |  |  |  |  |  |  |  |
|  | Regular employees | 13,926 | 8.1% | 1.00 |  | 323 | 13.9% | 1.00 |
|  | Part-timers & casual staff | 1009 | 8.9% | 1.12 (0.91–1.39) |  | 437 | 19.5% | 1.38 (0.98–1.93) |
|  | Other types of non-regular^b^ | 1471 | 10.5% | 1.15 (0.98–1.36) |  | 391 | 17.6% | 1.23 (0.87–1.76) |
|  | Self-employed | 3912 | 10.0% | 1.04 (0.79–1.36) |  | 1770 | 16.0% | 1.43 (0.87–2.38) |
|  | Non-working | 2323 | 12.6% | Not calculated |  | 5881 | 19.3% | Not calculated |
| Company size (number of employees) | | |  |  |  |  |  |  |
|  | ≥100 employees | 8912 | 7.8% | 1.00 |  | 376 | 21.0% | 1.00 |
|  | 1 to 4 employees | 4482 | 10.0% | 1.13 (0.87–1.47) |  | 1874 | 15.8% | 0.59 (0.35–1.0003) |
|  | 5 to 29 employees | 2850 | 8.7% | 1.04 (0.90–1.20) |  | 361 | 16.1% | 0.78 (0.57–1.07) |
|  | 30 to 99 employees | 2549 | 9.7% | 1.20 (1.04–1.38) |  | 247 | 17.8% | 0.91 (0.65–1.27) |
|  | Public servants | 1525 | 8.2% | 1.13 (0.93–1.37) |  | 63 | 6.3% | 0.28 (0.11–0.72) |
|  | Non-working | 2323 | 12.6% | Not calculated |  | 5881 | 19.3% | Not calculated |

CI, confidence interval; LBP, low back pain; PR, prevalence ratio.

^a^ Adjusted for age (per 5-year increase), marital status, family size, housing tenure, equivalent household expenditures, education, alcohol intake, smoking status, sleep duration, chronic medical conditions, and all three items of occupational class variables.

^b^ Temporary employees, contract staff, contract-based workers, and fixed-term employees.

**Supplementary Table 2** Stratified analyses by age group among women

|  |  | People aged 20–64 years (n = 24,634) | | |  | People aged 65 and older (n = 11,236) | | |
| --- | --- | --- | --- | --- | --- | --- | --- | --- |
|  |  | n | % LBP | PR^a^ (95% CI) |  | n | % LBP | PR^a^ (95% CI) |
| Occupation | |  |  |  |  |  |  |  |
|  | Clerks | 4081 | 9.2% | 1.00 |  | 179 | 14.5% | 1.00 |
|  | Managers | 289 | 10.0% | 0.96 (0.67-1.37) |  | 70 | 10.0% | 0.66 (0.30-1.47) |
|  | Professionals & technicians | 3894 | 11.4% | 1.24 (1.08-1.41) |  | 216 | 20.4% | 1.45 (0.93-2.27) |
|  | Sales | 1527 | 12.0% | 1.19 (1.002-1.41) |  | 160 | 22.5% | 1.43 (0.90-2.26) |
|  | Services | 3913 | 11.6% | 1.12 (0.98-1.28) |  | 444 | 17.6% | 1.13 (0.75-1.71) |
|  | Security/protective | 16 | 6.3% | 0.64 (0.09-4.52) |  | 0 |  |  |
|  | Agricultural/forestry/fishery | 266 | 17.3% | 1.69 (1.25-2.27) |  | 347 | 25.4% | 1.52 (0.999-2.30) |
|  | Manufacturing | 1074 | 11.6% | 1.11 (0.92-1.35) |  | 98 | 13.3% | 0.86 (0.46-1.60) |
|  | Transportation/machine | 26 | 11.5% | 0.91 (0.31-2.67) |  | 4 | 50.0% | 4.64 (1.79-12.00) |
|  | Construction/mining | 64 | 15.6% | 1.53 (0.84-2.80) |  | 14 | 7.1% | 0.45 (0.07-3.15) |
|  | Carrying/cleaning/packing | 424 | 15.6% | 1.37 (1.07-1.75) |  | 131 | 12.2% | 0.76 (0.42-1.36) |
|  | Other unclassified occupation | 653 | 11.6% | 1.13 (0.89-1.43) |  | 111 | 17.1% | 1.13 (0.66-1.94) |
|  | Non-working | 8407 | 11.3% | 1.21 (1.05-1.39) |  | 9462 | 21.3% | 1.68 (0.90-3.14) |
| Employment status | |  |  |  |  |  |  |  |
|  | Regular employees | 6171 | 9.8% | 1.00 |  | 126 | 12.7% | 1.00 |
|  | Part-timers & casual staff | 6065 | 12.1% | 1.12 (0.995-1.25) |  | 430 | 16.3% | 1.43 (0.86-2.39) |
|  | Other types of non-regular^b^ | 1606 | 11.5% | 1.10 (0.95-1.29) |  | 81 | 13.6% | 1.10 (0.55-2.19) |
|  | Self-employed | 2385 | 12.2% | 1.27 (0.98-1.64) |  | 1137 | 20.5% | 1.43 (0.73-2.81) |
|  | Non-working | 8407 | 11.3% | Not calculated |  | 9462 | 21.3% | Not calculated |
| Company size (number of employees) | | |  |  |  |  |  |  |
|  | ≥100 employees | 6439 | 11.1% | 1.00 |  | 178 | 16.3% | 1.00 |
|  | 1 to 4 employees | 3060 | 11.7% | 0.85 (0.67-1.08) |  | 1201 | 20.3% | 0.98 (0.53-1.84) |
|  | 5 to 29 employees | 3190 | 10.8% | 0.96 (0.85-1.09) |  | 221 | 14.9% | 0.86 (0.54-1.36) |
|  | 30 to 99 employees | 2475 | 10.9% | 0.97 (0.85-1.11) |  | 153 | 14.4% | 0.84 (0.51-1.40) |
|  | Public servants | 1063 | 11.3% | 1.09 (0.90-1.31) |  | 21 | 9.5% | 0.68 (0.17-2.71) |
|  | Non-working | 8407 | 11.3% | Not calculated |  | 9462 | 21.3% | Not calculated |

CI, confidence interval; LBP, low back pain; PR, prevalence ratio.

^a^ Adjusted for age (per 5-year increase), marital status, family size, housing tenure, equivalent household expenditures, education, alcohol intake, smoking status, sleep duration, chronic medical conditions, and all three items of occupational class variables.

^b^ Temporary employees, contract staff, contract-based workers, and fixed-term employees.
